# A Laterally Excited Bulk Acoustic Wave Resonator Based on LiNbO_3_ with Arc-Shaped Electrodes

**DOI:** 10.3390/mi15111367

**Published:** 2024-11-12

**Authors:** Jieyu Liu, Wenjuan Liu, Zhiwei Wen, Min Zeng, Chengliang Sun

**Affiliations:** 1 The Institute of Technological Sciences, Wuhan University, Wuhan 430072, China; jieyuuu@whu.edu.cn (J.L.); zwei_wen@whu.edu.cn (Z.W.); zengmm@whu.edu.cn (M.Z.); 2 Hubei Key Laboratory of Electronic Manufacturing and Packaging Integration, Wuhan University, Wuhan 430072, China; 3 Wuhan Institute of Quantum Technology, Wuhan University, Wuhan 430072, China

**Keywords:** XBAR, arc-shaped electrode, electromechanical coupling coefficient, spurious modes

## Abstract

High frequency and large bandwidth are growing trends in communication radio-frequency devices. The LiNbO_3_ thin film material is expected to become the preferred piezoelectric material for high coupling resonators in the 5G frequency band due to its ultra-high piezoelectric coefficient and low loss characteristics. The main mode of laterally excited bulk acoustic wave resonators (XBAR) have an ultra-high sound velocity, which enables high-frequency applications. However, the interference of spurious modes is one of the main reasons hindering the widespread application of XBAR. In this paper, a Z-cut LiNbO_3_ thin film-based XBAR with arc-shaped electrodes is presented. We investigate the electric field distribution of the XBAR, while the irregular boundary of the arc-shaped electrodes affects the electric field between the existing interdigital transducers (IDTs). The mode shapes and impedance response of the XBAR with arc-shaped electrodes and the XBARs with traditional IDTs are compared in this work. The fabricated XBAR on a 350 nm Z-cut LiNbO_3_ thin film with arc-shaped electrodes operating at over 5 GHz achieves a high effective electromechanical coupling coefficient of 29.8% and the spurious modes are well suppressed. This work promotes an XBAR with an optimized electrode design to further achieve the desired performance.

## 1. Introduction

With the advent of the 5G era, the demand for high-frequency and large-bandwidth communication equipment has surged, with stringent requirements for radio-frequency (RF) electronic components. In recent years, lithium niobate (LiNbO_3_) and lithium tantalate (LiTaO_3_) have attracted the attention of many researchers due to their large piezoelectric coefficients [1,2] and have been promising materials for high-band and large-bandwidth applications. Recently, with the development of smart cut technology, the application of LiNbO_3_ and LiTaO_3_ films in resonators have become possible. Thanks to thin film transfer technology, the field of resonators based on LiNbO_3_ and LiTaO_3_ thin films has attracted great attention in recent years [3,4,5,6,7,8,9,10,11], and they have been successfully applied to many types of resonators and filters. The laterally excited bulk acoustic wave resonator (XBAR) based on LiNbO_3_ and LiTaO_3_ thin films have the advantages of high frequency and a wide band, which can potentially meet the requirements of 5G high-frequency RF devices [12,13,14].

An effective electromechanical coupling coefficient ($K_{eff}^{2}$) is an important parameter to evaluate the conversion efficiency from electrical energy to mechanical energy of a resonator, which affects the bandwidth of subsequent filter applications. Michio Kadota and Takashi Ogami studied a lamb wave resonator using a Z-cut LiNbO_3_ thin crystal plate and found that it possessed a high series resonance frequency (*f_s_*) of 5.44 GHz and large $K_{eff}^{2}$ of 20.3% [15]. In 2019, Vicky Plessky et al. proposed a shear wave bulk acoustic resonator using a 400 nm thick ZY–LiNbO_3_ film resonating at 4.8 GHz, with a high $K_{eff}^{2}$ of 25% [16]. Yansong Yang et al. adopted interdigital transducers (IDTs), 500 nm in width, on a 500 nm thick Z-cut LiNbO_3_ thin film to fabricate a 4.5 GHz A1 mode resonator which had a $K_{eff}^{2}$ of 28% [17]. There are multiple factors affecting the $K_{eff}^{2}$ of resonators [18], such as electrode materials [18], piezoelectric materials [19], the geometric design of the electrodes [20], and the arrangement of the electrodes [21]. Chengliang Sun et al. proposed a checker-mode resonator, adopting checker-patterned electrodes to improve the $K_{eff}^{2}$ [22]. The design of the electrodes affects the distribution of the electric field thus influencing the piezoelectric effect and thereby changing the $K_{eff}^{2}$ [23]. While maintaining high electromechanical conversion efficiency, we also urgently need to address the issue of the influence of spurious modes on the performance of resonators.

In this work, we proposed a spurious mode suppression method by introducing arc-shaped electrodes to XBAR on a 350 nm Z-cut LiNbO_3_ thin film. The numerical simulations were carried out by the finite-element method (FEM) using COMSOL5.6 software. To analyze the suppression principle of the spurious modes, the mode shapes of the conventional XBAR with IDT electrodes were first simulated and analyzed. Then, the electric field distributions and impedance curves of the XBARs with two types of electrodes were studied and compared. In addition, we also conducted a parametric analysis on the short axis of the introduced arc-shaped electrode to verify its suppression effect on the spurious modes. Subsequently, we fabricated and tested the proposed XBAR with arc-shaped electrodes. The measurement results align well with the simulated results. The fabricated XBAR with arc-shaped electrodes displays a high resonant frequency of 5.2 GHz and high $K_{eff}^{2}$ of 29.8%, which exhibits a potential for high-frequency and broad-bandwidth applications.

## 2. Modeling and Principle

**Figure 1:**
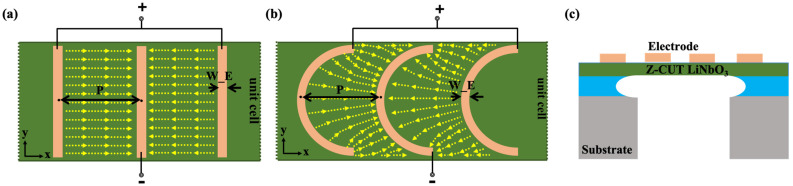
Two-dimensional cross-sectional view of XBARs (**a**) with IDT electrodes and (**b**) with arc-shaped electrodes. (**c**) The cross-sectional view of a XBAR based on a Z-cut LiNbO_3_ thin film.

The vertical views of the XBARs with IDT electrodes and with arc-shaped electrodes in the xy-plane are shown in Figure 1a,b, where the main geometric parameters of the resonator have been marked. *W_E* represents the width of the electrode. Pitch (*P*) is the center distance between adjacent electrodes. The cross-sectional diagram of XBAR based on a Z-cut LiNbO_3_ thin film is shown in Figure 1c. Similar to the structure of traditional bulk acoustic wave resonators, an electrode layer covers the piezoelectric layer, and a cavity is located below the piezoelectric layer in the active region of the resonator. Positive and negative voltages are alternately applied to the adjacent electrodes. The XBARs use the e_24_ and e_15_ piezoelectric coefficient of LiNbO_3_ to excite the horizontal displacements on the membrane surface. As the depth of the thin LiNbO_3_ membrane increases, the electric field strength gradually weakens.

**Figure 2:**
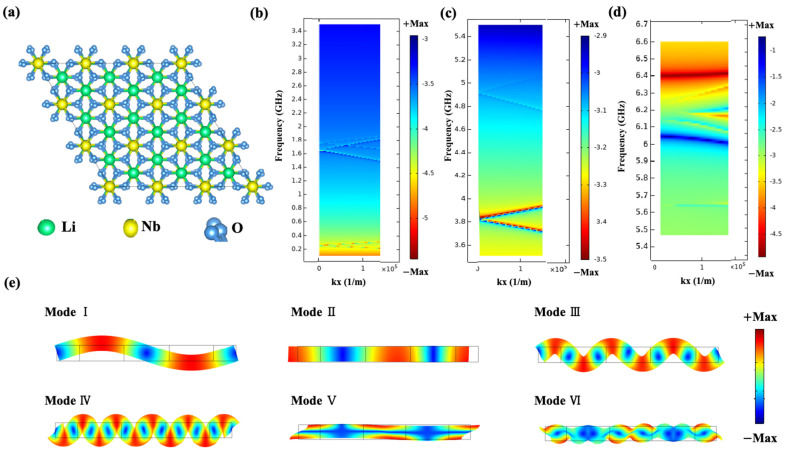
(**a**) The schematic of hexagonal unit cell of Z-cut LiNbO_3_. Dispersive diagrams for propagation on the x-axis for the real part of propagation constant (**b**) while the frequency domain is 0.1–3.5 GHz, (**c**) while the frequency domain is 3.5–5.5 GHz and (**d**) while the frequency domain is 5.5–6.5 GHz. (**e**) The total displacements of different mode shapes while kx = 0 (1/m).

In order to explore XBAR, research on the single-crystal lithium niobate piezoelectric materials were first conducted. As depicted in Figure 2a, a hexagonal unit cell of LiNbO_3_ material was analyzed. LiNbO_3_ crystals exhibit triple rotational symmetry around their c-axis. Therefore, it is a member of the trigonal crystal system. In addition, it exhibits mirror symmetry about three intersecting planes at 60 degrees, forming a triple axis of rotation. Therefore, LiNbO_3_ is classified as a 3 m point group and belongs to the R3c space group. In a triangular system, two completely different unit cells can be selected—hexagonal or rhombohedral. Z-cut LiNbO_3_ is widely used in high-frequency and wide-bandwidth devices due to its large piezoelectric coefficient. Dispersive diagrams for propagation on the x-axis for the real part of the propagation constant are shown in Figure 2b–d; the frequency ranges from 0.1 to 6.5 GHz. The numerical simulations were carried out by the two-dimensional finite-element method (FEM) using the COMSOL software. The main modes (SH_0_ mode, lateral vibrating mode, higher order SH modes, shear modes) in the Z-cut LiNbO_3_ thin film-based resonator can be observed in the figures below. Mode shape I in Figure 2e correspondes to the SH_0_ mode, which can be excited at low frequencies under certain specific conditions. Mode shape III and mode shape IV correspond to the higher order SH modes. Mode shape II represents the lateral vibrating mode, which mainly generates lateral horizontal displacements. Mode shape V corresponds to the A1 shear mode, while mode shape VI corresponds to the high order shear mode. The XBAR with a suspended Z-cut LiNbO_3_ membrane excited A1 mode vertical shear acoustic waves within the platelet.

## 3. Fabrication and Characterization

**Figure 3:**
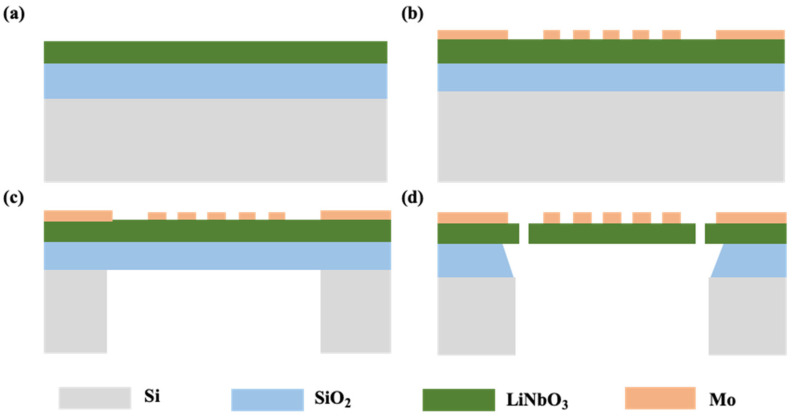
Process flows of XBAR: (**a**) The schematic diagram of LNOI wafer. (**b**) Definition of top electrode. (**c**) DRIE back surface Si. (**d**) Suspend the resonator using BOE.

The process flows are shown in Figure 3. As shown in Figure 3a, O_2_ plasma is adopted to clean the wafer’s surface. The Z-cut LiNbO_3_ on the insulator wafer has a 350 nm LiNbO_3_ piezoelectric layer and 2 μm silicon dioxide (SiO_2_). The top electrode is defined by sputtering 200 nm molybdenum (Mo) and then forming the pattern which shown in Figure 3b. Before employing the deep reactive ion etching (DRIE) technology, a layer of SiO_2_ is first deposited on top of the wafer to protect the electrodes. The silicon (Si) is directly removed via the DRIE method after patterning the upper Mo electrode and, finally, the remaining SiO_2_ is removed by buffered oxide etch (BOE) technology from the back of the device. We can control the verticality of the sidewalls after the DRIE process by adjusting the parameters of the DRIE process. Due to the chemical vapor phase reaction in the DRIE process, the side walls of the etched back cavity are also rough.

**Figure 4:**
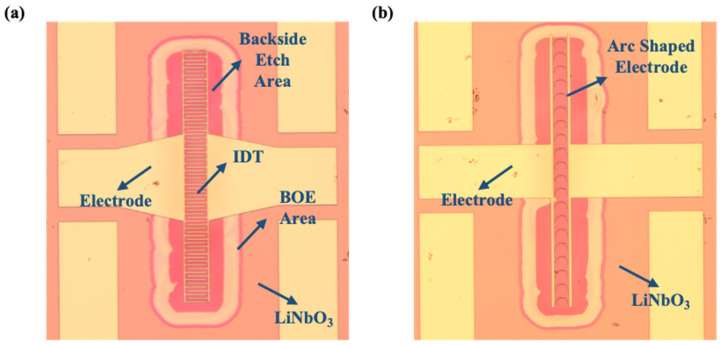
The microscope images of (**a**) device with IDT electrodes and (**b**) device with arc-shaped electrodes.

Figure 4a,b show the microscopic pictures of the fabricated XBARs with IDT electrodes and with arc-shaped electrodes, respectively. In the picture, the etched area of the back cavity is evenly distributed in the effective area of the device. The morphology of the whole device is acceptable. The film has no adhesion, and the metal is not oxidized. From the figure, it can be seen that the boundary after the BOE release method was performed is one circle larger than the boundary after DRIE process was performed, and it is very uniform. The active region of a resonator is defined by the boundary released by the BOE release method, so this needs to be taken into account when designing a resonator.

**Figure 5:**
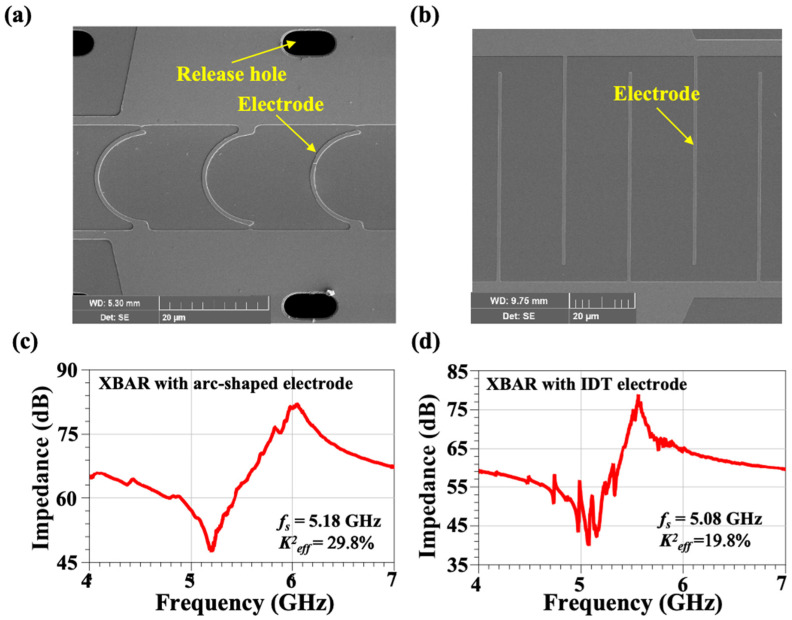
The SEM pictures of the fabricated XBARs (**a**) with arc-shaped electrodes and (**b**) with IDT electrodes. Measured impedance responses of XBARs (**c**) with arc-shaped electrodes and (**d**) with IDT electrodes.

This paper used a device-testing platform which mainly included a Cascade semi-automatic probe station, GSG RF probe, and Keysight network analyzer. The network analyzer was connected to the probe through a high-frequency transmission line. Figure 5a,b shows the SEM pictures of the fabricated XBARs with arc-shaped electrodes and with IDT electrodes, respectively. The geometric parameters corresponding to the resonator with IDT electrodes and with arc-shaped electrodes are listed in Table 1. The *P* in the XBARs with IDT electrodes and with arc-shaped electrodes is 20 μm and the *W_E* is 1 μm. The number of electrode pairs is defined as N, which is fixed at 20. The scattering parameter (S-parameters) measurement results of two port resonators with traditional arc-shaped electrodes and with IDT electrodes are shown in Figure 5c,d. The $K_{eff}^{2}$ in this work is defined by the following equation [24]:

(1) $$K_{eff}^{2}=\frac{\pi^{2}}{4}\frac{f_{s}(f_{p}-f_{s})}{f_{p}^{2}}$$

Among them, *f_s_* and *f_p_* correspond to the series resonant frequency and parallel resonant frequency of the resonator, respectively.

As shown in Figure 5c,d, compared with the performance of the XBAR with IDT electrodes, the XBAR with the arc-shaped electrodes exhibits a significant improvement in $K_{eff}^{2}$ and has better spurious mode suppression. The $K_{eff}^{2}$ of the XBAR with IDT electrodes is 19.8%, and there are many spurious modes. However, the spurious modes of the XBAR with arc-shaped electrodes are well suppressed. When the *W_E* is fixed at 1 μm, *P* is fixed at 20 μm, and *N* is fixed at 20, the $K_{eff}^{2}$ of the XBAR with arc-shaped electrode can reach 29.8%, which is much higher than that of the XBAR with IDT electrodes. Since the figure of merit (FOM) is about a fixed value, the XBAR with arc-shaped electrodes has a lower *Q* compared to that of the XBAR with IDTs.

## 4. Simulation and Analysis

**Figure 6:**
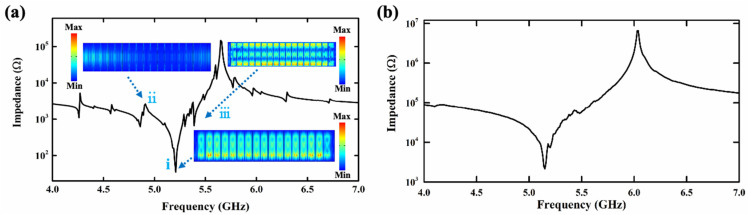
Simulated impedance curve of XBARs (**a**) with IDT electrodes and (**b**) with arc-shaped electrodes.

In order to further investigate the mechanism of the effect of arc-shaped electrodes on the performance of resonators, the three-dimensional FEM using COMSOL software is adopted in this article to simulate the frequency-domain characteristics of the resonators on suspended Z-cut LiNbO_3_ thin films. The simulated impedance curve of XBAR with traditional IDT electrodes is presented in Figure 6a. The resonator operates at 5.18 GHz, while the thickness of the LiNbO_3_ thin film is 350 nm and the thickness of the Mo electrode is 200 nm. Compared to the impedance curve of the XBAR with arc-shaped electrodes shown in Figure 6b, it can obviously be seen that there are many spurious modes in the impedance response of the XBAR with traditional IDT electrodes. To explore its principles, each spurious mode of the XBAR with traditional IDT electrodes was studied and analyzed. The mark (i) in Figure 6a corresponds to the main mode, which is an A1 shear mode. The spurious modes of the traditional XBAR mainly include ripples and transverse waves. As shown in Figure 6a, ripple (ii), caused by the resonance from the piezoelectric layer and electrodes, leads to XBARs with metal electrodes thus considering the metal as acoustically active. Therefore, its resonant frequency is lower than the main resonant frequency. Ripple (iii) is a spurious mode with standing waves in electrodes and transverse resonances.

**Figure 7:**
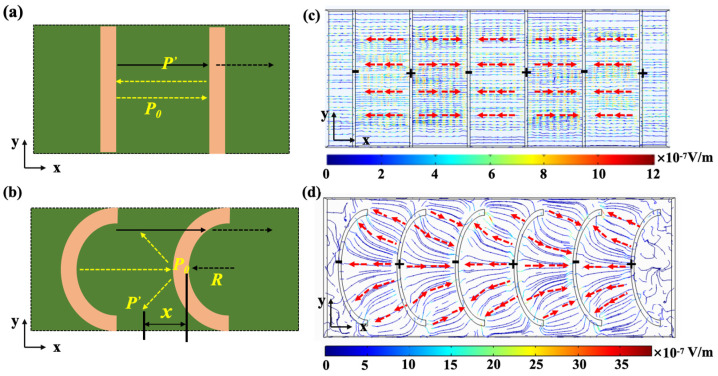
Schematic diagram of acoustic wave propagation between adjacent (**a**) IDT electrodes and (**b**) arc-shaped electrodes. Simulated electric field distributions of XBARs (**c**) with IDT electrodes and (**d**) with arc-shaped electrodes.

The spurious modes (ii) and (iii) are suppressed due to the introduction of arc-shaped electrodes, which can be observed in Figure 6b. The schematic diagrams of acoustic wave propagation between the adjacent IDT electrodes and between the arc-shaped electrodes are shown in Figure 7a and 7b, respectively. Since IDTs are placed in parallel, acoustic waves form agitation between the adjacent electrodes. The arc-shaped mode electrode has an irregular acoustic wave reflection boundary, which hardly forms agitation between the electrodes. The multipath reflection is conducive to the dissipation of non-main resonant acoustic waves. According to the following equation:

(2) $$P\prime =P_{0}\sqrt{0.5\times R/\left(x\pm 0.5\times R\right)}$$

the pressure of the reflected acoustic wave (*P′*) is reduced due to $\sqrt{0.5\times R/\left(x\pm 0.5\times R\right)}<1$, wherein the *x* is the distance from a point to irregular acoustic wave reflection boundary and the *R* is the curvature radius. The *P*_0_ is the incident wave sound pressure. It can be seen from Figure 7c,d that the electric field distributions of the XBARs with traditional IDT electrodes and arc-shaped electrodes are different. As shown in Figure 7, the electric field distributions between the traditional IDT electrodes are only distributed along the x-axis direction. However, the electric field distributions between the adjacent arc-shaped electrodes are irregular and divergent. From the figure, it can be seen that the introduction of arc-shaped electrodes can enhance the electric field, which is conducive to the conversion of electrical energy into mechanical energy, thereby improving the $K_{eff}^{2}$ of the resonator.

**Figure 8:**
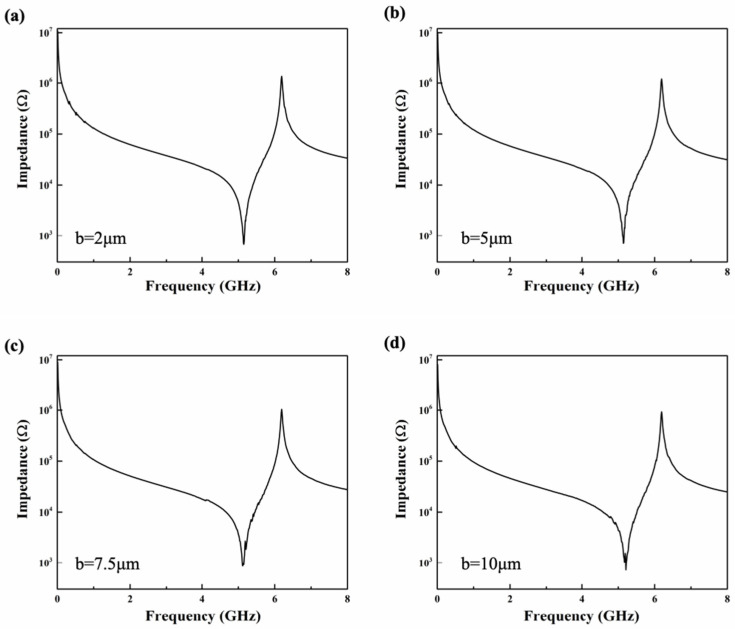
Impedance curve of arc-shaped electrode XBAR when a = 10 μm and (**a**) b = 2 μm; (**b**) b = 5 μm; (**c**) b = 7.5 μm; (**d**) b = 10 μm.

In addition, we investigated the effect of the ratio of the long axis (a) to the short axis (b) of the arc-shaped electrode on the performance of the resonator. In the simulation, to maintain a single variable, the piezoelectric layer thickness of the resonator is 0.35 μm, the electrode thickness is 0.2 μm, *P* is fixed at 20 μm, *W_E* is fixed at 1 μm, the long axis of the arc-shaped electrode is fixed at 10 μm, and a set of parameters (2 μm, 5 μm, 7.5 μm, 10 μm) are set for the short axis of the resonator. Figure 8 shows the impedance curves of the arc-shaped electrode XBAR under different short axes. As shown in Figure 8, when a remains constant, changing b has almost no effect on the *f_s_* and $K_{eff}^{2}$ of the resonator but has a certain impact on the ripple of the impedance curve. However, for the arc-shaped electrode XBAR under different short axes, the spurious modes are effectively suppressed, which also demonstrates the suppression effect of irregular electrode boundaries on the pseudo modes of resonators.

## 5. Conclusions

In this study, the arc-shaped electrodes are adopted on an XBAR to suppress the spurious modes and to improve the $K_{eff}^{2}$. The dispersive diagrams of the Z-cut LiNbO_3_ thin film-based resonator for its propagation on the x-axis for the real part of the propagation constant are studied. The spurious modes of the resonator are suppressed because the arc-shaped mode electrode has an irregular acoustic wave reflection boundary, which hardly forms agitation between the electrodes. The multipath reflection is conducive to the dissipation of non-main resonant acoustic waves. The electric field distributions of the XBAR with arc-shaped electrodes are more beneficial to improving the $K_{eff}^{2}$. The fabricated XBAR with arc-shaped electrodes, while *W_E* is 1 μm, *P* is 20 μm, and *N* is 20, demonstrates a high $K_{eff}^{2}$ of 29.8%, resonating over 5 GHz. This work provides us with a potential way to design and fabricate XBAR devices with enhanced performance.

## Figures and Tables

**Table 1:** Design Parameters of the Fabricated Resonators.

Parameter	XBAR with IDTs	XBAR with Arc-Shaped Electrodes
W_E	1	1
P	20	20
N	20	20
*f_s_* (GHz)	5.08	5.18
*f_p_* (GHz)	5.57	6.04
$K_{eff}^{2}$	19.8%	29.8%
*Q*	717	304

## Data Availability

The data presented in this study are available in this article.
